# Health policy implications for cardiovascular disease, type 2 diabetes mellitus, and stroke in Central Asia: a decadal forecast of their impact on women of reproductive age

**DOI:** 10.3389/fpubh.2024.1456187

**Published:** 2024-08-22

**Authors:** Sharapat Moiynbayeva, Valikhan Akhmetov, Nazerke Narymbayeva, Kuralay Shaikova, Dinara Makhanbetkulova, Magripa Bapayeva, Tamara Abdirova, Tatyana Popova, Indira Karibayeva

**Affiliations:** ^1^Department of Science and Consulting, Kazakhstan Medical University “KSPH”, Almaty, Kazakhstan; ^2^Department of Economics of Healthcare and Insurance Medicine, Kazakhstan Medical University “KSPH”, Almaty, Kazakhstan; ^3^Department of Public Health and Social Sciences, Kazakhstan Medical University “KSPH”, Almaty, Kazakhstan; ^4^Department of Nursing, Asfendiyarov Kazakh National Medical University, Almaty, Kazakhstan; ^5^Department of Internal Medicine, Kazakhstan Medical University “KSPH”, Almaty, Kazakhstan; ^6^Department of Health Policy and Community Health, Jiann-Ping Hsu College of Public Health, Georgia Southern University, Statesboro, GA, United States

**Keywords:** cardiovascular diseases, diabetes mellitus type 2, stroke, women’s health, forecast

## Abstract

**Introduction:**

Cardiovascular disease, type 2 diabetes, and stroke are significant global health concerns. However, gaps persist in understanding the impact of these disorders on women of reproductive age in Central Asia. This study aimed to analyze the health policies implemented in Central Asian countries to address the healthcare needs of this demographic and to forecast future trends in prevalence rates.

**Methodology:**

We forecasted future trends in prevalence rates, years of life lost, years lived with disability, and disability-adjusted life years for cardiovascular disease, type 2 diabetes, and stroke using publicly available data. Two data sources were utilized: health policy documents issued by the governments of Kazakhstan, Kyrgyzstan, Uzbekistan, Tajikistan, and Turkmenistan, and data from the Institute for Health Metrics and Evaluation. Forecasting models, including ARIMA, were employed to predict trends until 2030.

**Results:**

The results indicate an anticipated increase in cardiovascular disease prevalence from 1856.55 in 2020 to 2007.07 by 2029 in Kazakhstan, a subtle increase in Kyrgyzstan from 2492.22 to 2558.69 over 10 years, and similar trends in other countries.

**Conclusion:**

The analysis of policy documents revealed a lack of specific focus on addressing cardiovascular disease, stroke, or type 2 diabetes outside the contexts of pregnancy and childbirth. Understanding these trends is crucial for informing targeted health interventions and resource allocation to mitigate the impact of these diseases on women’s health in Central Asia.

## 1 Introduction

The Central Asian region, comprising Kazakhstan, Kyrgyzstan, Uzbekistan, Tajikistan, and Turkmenistan, holds significant strategic importance due to its geographic location in the heart of the Eurasian continent and its abundant natural resources. These countries gained independence in 1991 following the dissolution of the Soviet Union, leading to a socio-economic crisis as they transitioned their national economies from the Soviet planned economy to a capitalist model (1). They inherited the Semasko model of healthcare, which emphasized the control of communicable diseases, maternal, and child health. Presently, these countries share many similarities in healthcare governance, budgeting, and the prioritization of health issues (2).

Central Asian countries traditionally experience high birth rates, exceeding those observed in their former Soviet bloc neighbors. Similarly, they have historically faced elevated rates of maternal mortality, particularly during the early transition period (3). For instance, Kyrgyzstan exhibited the highest maternal mortality rate in the entire World Health Organization (WHO) European region (4). Suboptimal perinatal care contributed to this phenomenon, prompting Central Asian countries to focus on maternal health as a strategic goal. These efforts have led to a significant improvement in maternal mortality rates, although they remain higher than those observed in other countries belonging to the WHO European region (5). Central Asian countries continue to focus on maternal health while paying less attention to non-communicable diseases affecting women in their reproductive years. However, to further reduce maternal mortality, there is a need to address the issue of non-communicable diseases in this demographic (6).

Non-communicable diseases, including cardiovascular disease (CVD) and Type 2 Diabetes Mellitus (DM2), have emerged as global health concerns, affecting a considerable portion of the population. Although these disorders traditionally affect older age groups, they are increasingly prevalent among younger populations (7). Major types of cardiovascular disease include ischemic heart disease (IHD), heart failure (HF), stroke, coronary artery disease (CAD), and peripheral artery disease, which can lead to complications with potentially lethal outcomes if not timely diagnosed and adequately treated (8). There is a lack of scientific research investigating the impact of CVD and DM2 on women of reproductive age residing in Central Asia, as well as a dearth of research focusing on health policies created in Central Asian countries to address the health needs of these women. This data is crucial for better tailoring healthcare services to the needs of this population group. Therefore, this study aims to analyze the health policies implemented in Central Asian countries to address the health needs of women of reproductive age and to forecast future trends in changes in prevalence rates, Years of Life Lost (YLL), Years Lived with Disability (YLD), and Dis-ability-Adjusted Life Years (DALY) for CVD, DM2, and stroke using publicly available data. The hypothesis of the study is that non-communicable diseases such as CVD and DM2 significantly impact women of reproductive age in Central Asia, and there is a lack of comprehensive health policies addressing these diseases outside the context of pregnancy and childbirth.

## 2 Materials and methods

### 2.1 Data sources

Two data sources were utilized in this study. The first data source consisted of health policy documents governing the provision of healthcare services to women of reproductive age issued by the governments of Kazakhstan, Kyrgyzstan, Uzbekistan, Tajikistan, and Turkmenistan. Searches were conducted using the Google search engine, employing keywords such as “охрана здоровья” (health protection), “оказание медицинской помощи” (provision of medical care), “женщины репродуктивного возраста” (women of reproductive age), “сердечно-сосудистые заболевания” (cardiovascular disease), “сахарный диабет” (diabetes mellitus), “инсульт” (stroke), “Казахстан” (Kazakhstan), “Кыргызстан” (Kyrgyzstan), “Узбекистан” (Uzbekistan), “Таджикистан” (Tajikistan), and “Туркменистан” (Turkmenistan). All searches were conducted in the Russian language, as it is the official language in the countries of Central Asia (9). Google was chosen due to its widespread use, and prior to the search, browser history and cookies were cleared to avoid personalized search results based on previous activity. The first 50 search results were evaluated for each query.

Inclusion criteria encompassed legislative acts pertaining to the provision of care to women of reproductive age in the aforementioned Central Asian countries, while exclusion criteria involved legislative acts beyond the scope of this study. A table summarizing the key provisions of these legislative acts was compiled.

The second data source utilized in this study was sourced from the Institute for Health Metrics and Evaluation (IHME). As a globally recognized institution, IHME offers a comprehensive repository of health-related data from various regions world-wide, including Central Asian countries. Data covering the period 1991–2019 (from the dissolution of the Soviet Union to the latest available year) were obtained from the official website (10). Information pertaining to the prevalence rates, YLL, YLD, and DALY for CVD, DM2, and stroke was extracted. Any anomalies or inconsistencies in the data were addressed through cross-referencing with supplemental materials on the Global Burden of Disease Study (11) to ensure data accuracy.

### 2.2 Terminologies used

This study utilized the following terminologies:

- Reproductive age women: Defined as women aged 15–49 years (12).
- Prevalence rate: Defined as the proportion of individuals affected by a specific disease (13).
- Years of life lost (YLL): Defined as the years of potential life lost due to premature deaths (14).
- Years lived with disability (YLD): Defined as the years lived in a state of health less than ideal (14).
- Disability-adjusted life years (DALYs): Defined as the loss equivalent to 1 year of full health. DALYs represent the sum of YLL and YLD (14).

### 2.3 Statistical analysis

The extracted data on the prevalence rates, years of life lost (YLL), years lived with disability (YLD), and disability-adjusted life years (DALY) of cardiovascular disease (CVD), stroke, and Type 2 diabetes mellitus (DM2) were entered into an Excel spreadsheet. All statistical analyses were conducted using the Statistical Package for Social Sciences (SPSS) software, version 20. To project future values for prevalence rates, YLLs, YLDs, and DALYs, the Expert Modeller function of SPSS was utilized to retrieve the best-fit ARIMA (Autoregressive Integrated Moving Average) model. The ARIMA modeling technique was selected due to its known precision in handling time-series data, enabling the forecasting of future trajectories with a considerable degree of confidence based on historical trends (14). Key findings and trends were then visualized using graphical representations generated by the SPSS software, depicting past trends for the period 1991–2019 and future projections until 2030, along with their 95% confidence intervals.

## 3 Results

### 3.1 Analysis of policy documents governing the provision of healthcare services for women of reproductive age in Central Asian countries

Table 1 provides an overview of the policy documents adopted in the countries of Central Asia subsequent to the dissolution of the Soviet Union, aimed at regulating the provision of healthcare services to women of reproductive age. These policies primarily emphasized the enhancement of pregnancy-and childbirth-related care, the provision of contraception and family planning services, and the prevention of HIV and sexually transmitted diseases (STD). It was only in the proximity of the 2020s that policymakers began addressing other aspects of women’s health, broadly categorizing them as “extragenital pathologies.” In Uzbekistan, particular attention was given to the treatment of women with iron-deficiency anemia and the provision of premarital screening. However, there is a lack of policy documents in open access for Turkmenistan, which hinders the analysis of measures implemented for the provision of healthcare services to women of reproductive age. It is noteworthy that there was a lack of specific focus on addressing CVD, stroke, or DM2 among women of reproductive age outside the contexts of pregnancy and childbirth.

**Table 1:** Provision of healthcare services to women of reproductive age.

Country	Title of the policy document, year of adoption	Main provisions
Kazakhstan	State Law “On protecting the health of citizens,” 1997 (15)	The Law ensures the provision of perinatal and neonatal services at the expense of the state budget.
	State Law “On the reproductive rights of citizens and guarantees of their implementation,” 2004 (16)	The Law envisages the provision of reproductive and obstetrical services.
	MoH* Order “On measures to develop reproductive health care for citizens and provide family planning services,” 2009 (17)	The Order envisages measures directed at reducing the number of abortions and maternal mortality.
	State Law “On people’s health and healthcare system,” 2020 (18)	The Law specifies the provision of reproductive services to the general population and certain population groups. It also ensures the provision of treatment for major diseases that directly affect women’s reproductive health, funded by the state budget.
Kyrgyzstan	State Law “On reproductive rights of citizens,” 2000 (19)	This law established state guarantees for the protection of reproductive health and created a legal basis for the provision of family planning services
	MoH Order “On the implementation of the “Jean-ene” program (improving perinatal care) for 2002–2006 in the Kyrgyz Republic,” 2002 (20)	The Order envisages the provision of perinatal care services aimed at reducing maternal mortality.
	National Strategy “For the protection of reproductive health of the population of the Kyrgyz Republic until 2015,” 2006 (21)	The strategy regulates the provision of reproductive services to the general population and certain population groups, and sets preventive strategies against reproductive system cancers, HIV and STD**.
	MoH Order “On improving the organization of reproductive health services for children, adolescents, and young people in the Kyrgyz Republic.,” 2011 (22)	The Order envisages the provision of reproductive services for adolescents and young people.
	State Law “On protecting the health of citizens in the Kyrgyz Republic,” 2024 (23)	The Law specifies the provision of reproductive services. It also ensures the provision of treatment for major diseases that directly affect women’s reproductive health, funded by the state budget.
Uzbekistan	The Cabinet of Ministers’ Resolution “On the state program of measures for 1999 to strengthen the role of women in the family, state and social construction, and to improve the system for protecting their legal, social, economic interests,” 1999 (24)	The Resolution stipulates that conditions will be created to ensure the strengthening of the health of mothers and children, and the development of physical culture.
	The Cabinet of Ministers’ Resolution “On measures to implement priority directed towards increasing medical culture in the family, strengthening women’s health, and promoting the birth and raising of a healthy generation,” 2002 (25)	The Resolution envisages the equipping of obstetrics facilities with modern equipment, early detection of extragenital diseases, and providing all pregnant women with anemia with iron-containing medications.
	President’s Resolution “On the State Program ‘Year of a Healthy Mother and Child,’” 2016 (26)	The Resolution envisages measures to improve the equipping and staffing of rural healthcare facilities, stipulates the provision of full premarital medical examination, and promotes the widespread popularization of physical culture and sports among young girls.
	President’s Resolution “On measures to protect the health of mothers and children, and to strengthen the reproductive health of the population,” 2023 (27)	The Resolution envisages screening of pregnant women for obstetrical and genetic pathologies and stipulates measures for the prevention and management of extragenital diseases among the population of reproductive age.
Tajikistan	State Law “On reproductive health and reproductive rights,” 2002 (28)	The Law envisages the provision of reproductive and obstetrical services.
	Government’s Decree “On approval of the Strategic Plan of the Republic of Tajikistan on reproductive health of the population for the period until 2014,” 2004 (29)	The Decree envisages measures directed at the provision of contraception and family planning services, the reduction of perinatal and maternal mortality, the prevention of HIV and STDs, and screening for breast cancer.
	State reproductive health program for 2019–2022, 2019 (30)	The program stipulates measures to increase the availability and effectiveness of reproductive services, equip reproductive facilities with modern equipment, and distribute contraceptives.
	Government’s Resolution “On state reproductive health program for 2023–2027,” 2023 (31)	The program serves as a continuation of the 2019–2022 program and sets the same goals.
Turkmenistan	State Law “On protecting the health of citizens,” 2015 (32)	The Law envisages the provision of healthcare services, including reproductive health.

*MoH, Ministry of Health, **STD, sexually transmitted disease.

### 3.2 Cardiovascular disease, diabetes mellitus type 2, and stroke forecasting in women of reproductive age

The analysis of health policy documents revealed a lack of strategies directed at the prevention and treatment of CVD, DM2, and stroke in women of reproductive age residing in Central Asia. Consequently, the next phase of our study applied forecasting methods to predict the expected rates of these disorders over the next decade. This step is essential for evaluating the necessity of formulating targeted interventions aimed at reducing the burden of CVD, DM2, and stroke in this particular category of women.

#### 3.2.1 Forecasting of prevalence rates

**Figure 1:**
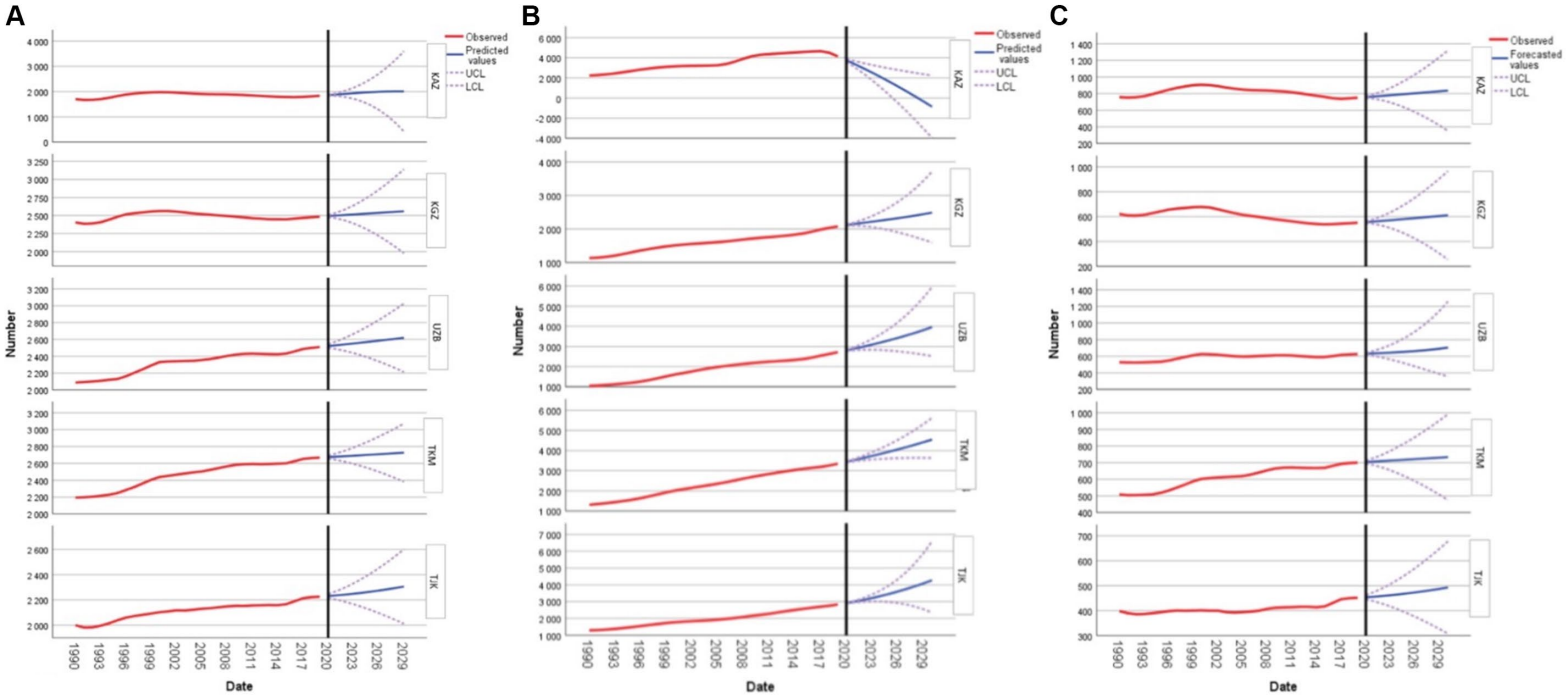
Forecasting cardiovascular disease, type 2 diabetes mellitus, and stroke prevalences in women of reproductive age: **(A)** forecasting cardiovascular disease; **(B)** forecasting type 2 diabetes mellitus; and **(C)** forecasting stroke for Kazakhstan, Kyrgyzstan, Uzbekistan, Tajikistan, and Turkmenistan.

In Kazakhstan, the forecasted values of CVD prevalence rate depict a continuous rise, starting at 1856.55 in 2020 and culminating at 2007.07 by 2029, suggesting an in-creasing health burden over the decade. In Kyrgyzstan, the values reveal a subtle up-ward trend, moving from 2492.22 in 2020 to 2558.69 in 2029, indicating a slower rate of growth in the projected health metrics compared to Kazakhstan. Uzbekistan presents a consistent increment in the forecasted values, with numbers starting at 2520.29 in 2020 and reaching 2619.48 by 2029. This progression mirrors that of Kazakhstan, albeit at a slightly lower magnitude. Turkmenistan’s projections show a steady increase from 2674.02 in 2020 to 2727.37 in 2029, representing a growth rate similar to that of Kyrgyzstan. Lastly, Tajikistan exhibits a moderate upward trajectory, with forecasted values growing from 2230.75 in 2020 to 2306.43 by 2029 (Figure 1A).

Regarding DM2, the data for Kazakhstan demonstrate a decline in the forecasted values over the decade, starting from 3758.09 in 2020 and decreasing to-857.42 by 2030, suggesting a potential decrease in DM2 prevalence in the future. Kyrgyzstan’s forecasted values indicate a consistent upward trend. The prevalence rate is expected to rise from 2115.88 in 2020 to 2487.98 by 2030, revealing a steady increase in projected DM2 cases. Uzbekistan’s projections exhibit a strong positive trend. Starting at 2806.58 in 2020, the values are anticipated to rise to 3960.96 by 2030, reflecting a significant growth in DM2 prevalence. Turkmenistan’s forecasted figures present a steady in-crease from 3437.86 in 2020 to 4543.86 by 2030, emphasizing a continuous upward trend in DM2 cases. For Tajikistan, the forecasted values suggest a consistent rise in DM2 prevalence, moving from 2912.90 in 2020 to 4272.31 by 2030 (Figure 1B).

Forecasting the prevalence rate of stroke in women of reproductive age reveals the following trends for the years 2020 to 2030: In Kazakhstan, forecasted values demonstrate a consistent increase from 757.62 in 2020 to 835.94 in 2030. Kyrgyzstan’s projections initiate at 555.67 in 2020, rising to 610.58 by 2030 at a somewhat slower rate. Uzbekistan’s data starts at 631.04 in 2020, escalating to 703.64 by 2030 with a notable acceleration in the latter years. Turkmenistan, with relatively higher initial values at 703.47 in 2020, sees a minimal increment, reaching 733.32 by 2030. Lastly, Tajikistan’s values, commencing at a lower 453.68 in 2020, experience a moderate rise to 492.89 by 2030 (Figure 1C).

#### 3.2.2 Forecasting of YLLs

**Figure 2:**
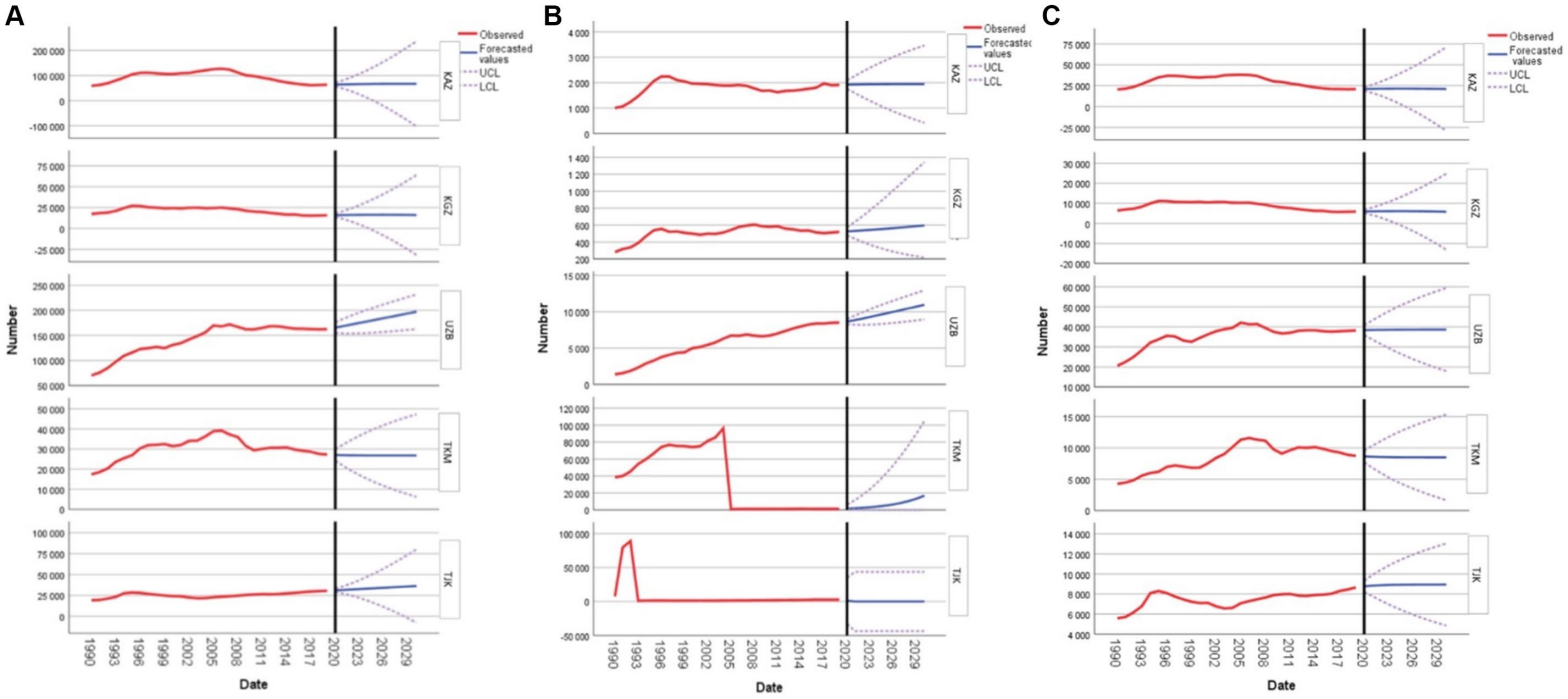
Forecasting cardiovascular disease, type 2 diabetes mellitus, and stroke years of lost life in women of reproductive age: **(A)** forecasting cardiovascular disease; **(B)** forecasting type 2 diabetes mellitus; and **(C)** forecasting stroke for Kazakhstan, Kyrgyzstan, Uzbekistan, Tajikistan, and Turkmenistan.

In Kazakhstan, there is a discernible increase in YLL due to CVD from 63,712.26 in 2020 to 66,804.95 by 2030, signaling sustained concern for cardiovascular health. Kyrgyzstan mirrors this trend with a rise from 15,886.38 in 2020 to 16,062.62 by 2030, albeit at a more modest rate. A more pronounced surge is observed in Uzbekistan, where the YLL value begins at a significant 165,480.55 in 2020 and is projected to reach an alarming 197,198.20 by 2030. Contrarily, Turkmenistan’s trajectory is slightly regressive, with YLL values decreasing minimally from 27,089.74 in 2020 to 26,810.04 by 2030. Lastly, Tajikistan’s forecast suggests a rise in YLL values over the decade. Collectively, these projections underscore the varying magnitudes of cardiovascular health challenges faced by women in the reproductive age group across these nations (Figure 2A).

Regarding DM2, the forecasted YLL values for Kazakhstan depict a subtle increment, starting from 1,923.36 in 2020 and reaching 1,945.68 by 2030. Kyrgyzstan’s YLL values also exhibit a steady rise, beginning at 526.09 in 2020 and climbing to 596.33 by 2030. Uzbekistan’s figures show a more pronounced increase, with YLL values commencing at 8,653.49 in 2020 and soaring to 10,953.08 by 2030. In stark contrast, Turkmenistan experiences an exponential surge, with YLL beginning at 1,611.05 in 2020 and skyrocketing to 16,875.32 by 2030. Notably, the data for Tajikistan presents an anomaly with an initial YLL value of 1,031.99 in 2020 but no further values provided for the subsequent years. These projections emphasize the diverse challenges posed by DM2 among women in the reproductive age group in these nations (Figure 2B).

Figure 2C provides insights into the decade-long projection of YLL due to strokes in women aged 15 to 49 years, spanning from 2020 to 2030. For Kazakhstan, the forecasted YLL starts at 21,019.31 in 2020, gradually increasing every year to reach its peak at 21,368.20 in 2025. After this, there is a consistent decline, ending at 20,979.87 in 2030. In Kyrgyzstan, the numbers begin at 5,945.32 in 2020, rise slowly, peaking at 6,086.38 in 2024, and then taper off slightly to 5,806.94 by 2030. For Uzbekistan, the YLL begins at 38,374.77 in 2020 and experiences minor fluctuations before settling at 38,668.07 in 2030. Turkmenistan’s numbers start at 8,639.07 in 2020, with minor fluctuations throughout the decade, finally resting at 8,495.89 in 2030. Lastly, Tajikistan’s forecast-ed YLL begins at 8,769.22 in 2020, slowly rises to 8,946.30 in 2030, reflecting a steady but moderate increase over the decade.

#### 3.2.3 Forecasting of YLDs

**Figure 3:**
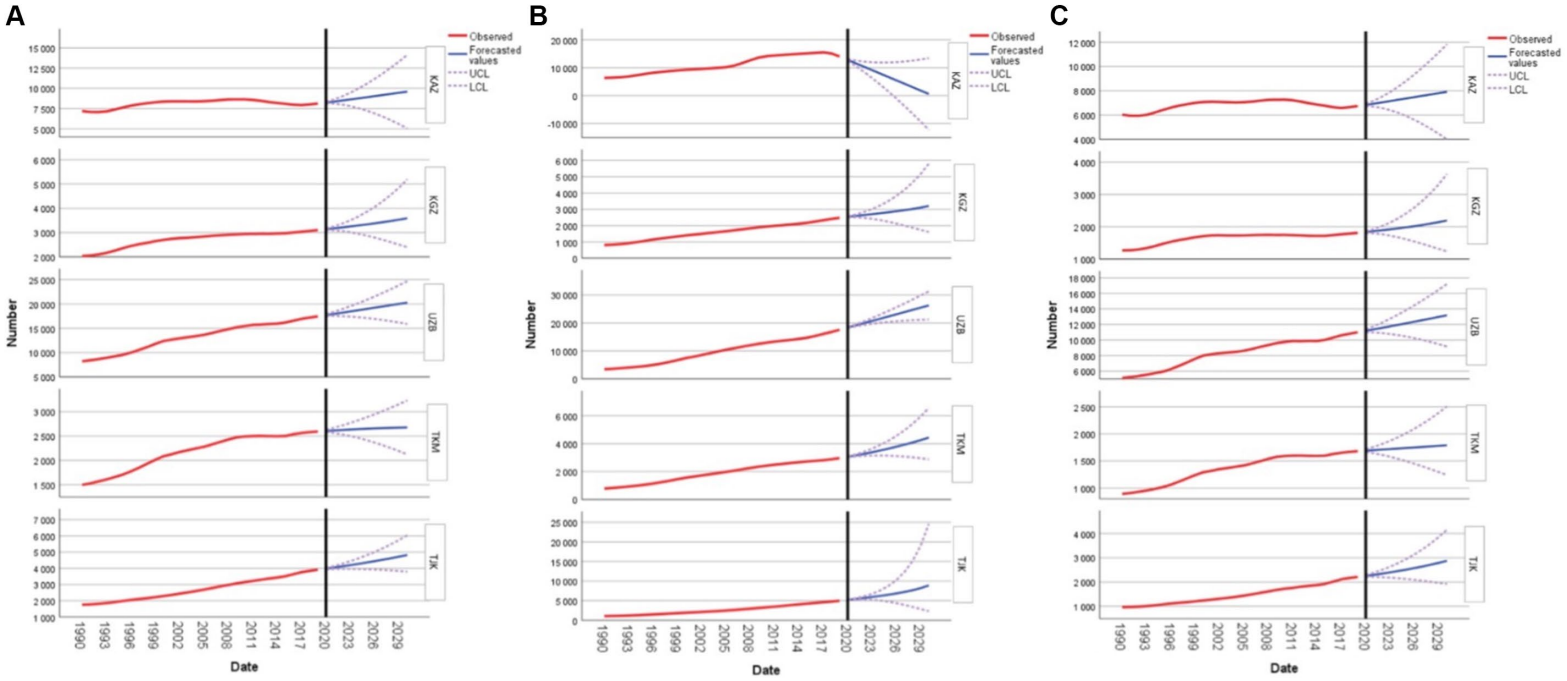
Forecasting cardiovascular disease, type 2 diabetes mellitus, and stroke years lived with disability in women of reproductive age: **(A)** forecasting cardiovascular disease; **(B)** forecasting type 2 diabetes mellitus; and **(C)** forecasting stroke for Kazakhstan, Kyrgyzstan, Uzbekistan, Tajikistan, and Turkmenistan.

Forecasting of YLD due to CVD in women of reproductive age in Kazakhstan indicates a systematic increase, starting at 8,242.04 in 2020 and reaching 9,591.68 by 2030. Kyrgyzstan’s figures begin at 3,143.07 in 2020, demonstrating a consistent rise to 3,593.46 by the end of the decade. In comparison, Uzbekistan’s projections start at a significantly higher value of 17,713.77 in 2020, climbing to an impressive 20,285.72 by 2030. Conversely, Turkmenistan’s forecasted figures indicate a more modest ascent, from 2,606.56 in 2020 to 2,678.54 by 2030. Concurrently, Tajikistan’s data sets out from 3,979.45 in 2020, with a pronounced upward trend concluding at 4,816.89 by the decade’s end (Figure 3A).

As for DM2, in Kazakhstan, the forecasted YLD values show a decreasing trend. Starting at 12,794.14 in 2020, there is a notable decline each year, reaching a value of 611.82 by 2030. This indicates a significant improvement in the health metrics for women in this age group in Kazakhstan over the decade. Kyrgyzstan presents a different scenario. The YLD values are forecasted to rise gradually over the years. The starting value in 2020 is 2,541.20, and by 2030, it is expected to reach 3,203.86. This suggests that there might be growing health challenges for women in Kyrgyzstan in the coming years. In Uzbekistan, the YLD values are also on the rise. Starting at 18,358.38 in 2020, the values are projected to increase steadily, reaching 26,275.28 by 2030. This consistent increase indicates that the health conditions for women in this age group in Uzbekistan might be deteriorating. Turkmenistan also exhibits a growing trend in YLD values. Beginning at 3,056.22 in 2020, the values are anticipated to climb, reaching 4,433.09 by 2030. This upward trajectory suggests potential health challenges for women in Turkmenistan. Lastly, Tajikistan reveals a significant increase in the YLD values over the forecasted period. From a value of 5,179.76 in 2020, there is a steep rise, culminating at 8,876.54 by 2030. This rapid increase might be indicative of emerging health issues for women in Tajikistan (Figure 3B).

The differences in YLD values between these countries highlight the varied im-pacts of stroke on women’s health. In Kazakhstan, the forecasted YLD values for this demographic show a steady increase. Starting from 6,839.93 in 2020, the figures are projected to reach 7,918.11 by 2030. Similarly, Kyrgyzstan reveals a consistent uptick, with YLD values beginning at 1,837.23 in 2020 and anticipated to climb to 2,193.26 by 2030. Uzbekistan, on the other hand, demonstrates a more pronounced growth. Here, the YLD values for these women are expected to initiate at 11,184.14 in 2020 and rise sharply to 13,188.38 by 2030. Notably, the steeper ascent in Uzbekistan’s figures stands out, emphasizing the increasing burden of stroke in the country (Figure 3C).

#### 3.2.4 Forecasting of DALYs

**Figure 4:**
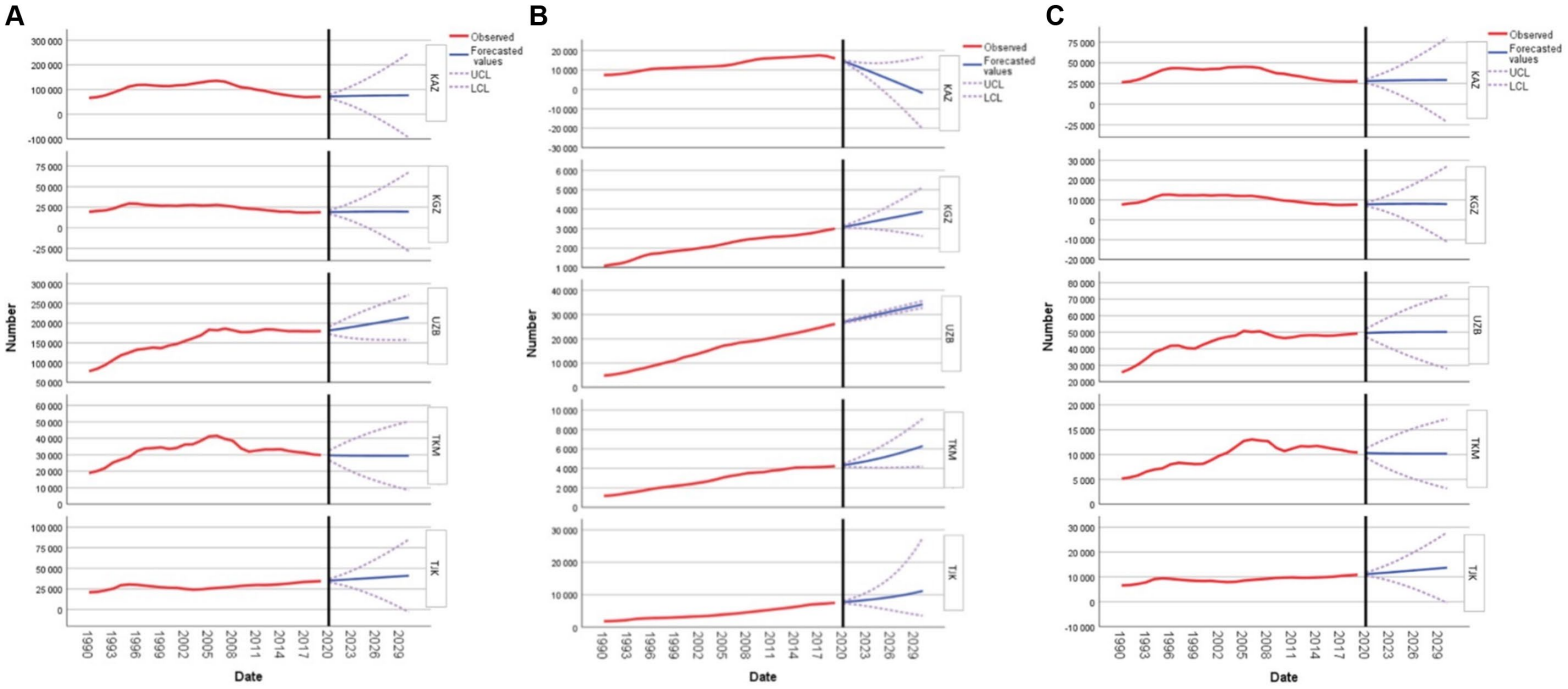
Forecasting cardiovascular disease, type 2 diabetes mellitus, and stroke disability-adjusted life years in women of reproductive age: **(A)** forecasting cardiovascular disease; **(B)** forecasting type 2 diabetes mellitus; and **(C)** forecasting stroke for Kazakhstan, Kyrgyzstan, Uzbekistan, Tajikistan, and Turkmenistan.

In Kazakhstan, the DALY values for CVD are witnessing a gradual upswing. Starting from 71,950.04 in 2020, these values are projected to reach 76,612.11 by 2030. This consistent rise suggests that Kazakhstan may need to intensify its healthcare interventions and awareness campaigns targeting CVD in women of reproductive age. Kyrgyzstan’s numbers showcase a modest increase, moving from 19,028.69 in 2020 to 19,583.64 by 2030. While the rise is not as pronounced as in some other nations, it still points to a growing concern that requires attention. On the other hand, Uzbekistan’s forecasted figures are particularly alarming, with projections escalating from 181,651.20 in 2020 to a significant 214,579.21 by 2030. This marked increase is the steepest among the countries listed and highlights an urgent need for robust public health measures in Uzbekistan. Interestingly, Turkmenistan presents a contrasting picture with a slight decline in DALY values, descending from 29,689.99 in 2020 to 29,410.37 by 2030. This decrease, though marginal, is a positive sign and could be indicative of effective measures already in place or perhaps different health dynamics within the country. Meanwhile, the projections for Tajikistan reveal a consistent growth trajectory, with values rising from 35,104.85 in 2020 to 41,071.51 by 2030, emphasizing the need for targeted health initiatives. Both Uzbekistan, with its sharp in-cline, and Turkmenistan, with its decline, stand out and offer valuable insights for further research and policy formulation in the region (Figure 4A).

In Kazakhstan, the estimated DALY for DM2 in 2020 is 14,561.30, which intriguingly reduces to-1,899.74 by 2030. Kyrgyzstan shows an increase from 3,075.86 in 2020 to 3,865.12 in 2030. Uzbekistan’s numbers are projected to rise from 26,824.25 in 2020 to 34,152.06 in 2030. Similarly, in Turkmenistan, the figures indicate a compelling upward trend from 4,334.11 in 2020 to 6,292.16 by 2030. Lastly, Tajikistan’s estimates for women in this age group, starting at 7,718.02 in 2020, are also anticipated to climb to 11,168.28 in 2030 (Figure 4B).

For Kazakhstan, the projected DALY due to stroke values show a gradual increase from 27,856.61 in 2020 to 29,110.73 by 2030. Similarly, in Kyrgyzstan, there is a modest growth in DALY values from 7,782 in 2020 to 7,930.37 in 2030. Uzbekistan’s projections start from 49,507.68 in 2020, slightly increasing to 50,147.05 by 2030. Turkmenistan, on the other hand, presents a minor decrease in DALY, starting from 10,323.58 in 2020 and settling at 10,179.45 by 2030. Lastly, Tajikistan’s values are on the rise, starting from 11,102.41 in 2020 and culminating at 13,713.74 by 2030 (Figure 4C).

## 4 Discussion

This study examines the health policies enacted in Central Asian nations to cater to the healthcare needs of women in their reproductive age. Addition-ally, it aims to predict forthcoming trends concerning changes in prevalence rates, YLLs, YLDs, and DALYs for CVD, DM2, and stroke, utilizing publicly available data. By delving into this demographic, the study seeks to provide invaluable insights into the health policies implemented in Kazakhstan, Kyrgyzstan, Uzbekistan, Tajikistan, and Turkmenistan, while aligning and enhancing the available information with potential impacts.

The analysis draws upon official documents and open-source data to substantiate its findings. By focusing on these five Central Asian countries, the study sheds light on the approaches taken by policymakers to address the healthcare needs of women of reproductive age. It underscores that while these nations have implemented various strategies, their attention has predominantly centered around issues related to pregnancy and childbirth. Only in recent years has there been a nascent focus on addressing “extragenital pathologies,” albeit in broadly defined terms.

The forecast modeling predicts a concerning rise in CVD and stroke prevalence rates and YLDs across all studied nations over the next decade. This emphasizes the increasing burden of CVD and stroke in the region, underlining the urgent need for targeted interventions and comprehensive health policies. For DM2, all countries, except Kazakhstan, are expected to experience increases in prevalence rates, YLDs, and DALYs. These findings represent a pivotal guide for policymakers, furnishing insights into the evolving health dynamics of Central Asia and underscoring the imperative of proactive measures to mitigate the burgeoning health challenges, particularly those concerning the specified diseases that impact women of reproductive age.

Our findings underscore the importance of implementing comprehensive preventive healthcare programs tailored to the needs of women in their reproductive years (33). Implementing comprehensive preventive healthcare programs specifically tailored to the needs of women in their reproductive years holds immense promise in curtailing the incidence of these ailments. These programs should encompass strategies targeting primary, secondary, and tertiary prevention. Primary prevention initiatives entail modifying health behaviors, such as promoting healthy dietary habits and physical activity (34). Despite the existence of programs promoting physical exercise in many countries of the region, their focus tends to be on children, adolescents, and young adults, neglecting other demographic groups (15, 23, 32). Moreover, there is a pressing need for health education campaigns and life-style interventions addressing risk factors like unhealthy dietary patterns and stress. A prior study revealed that dietary practices in certain countries under scrutiny, such as Kazakhstan, are notably poor, characterized by excessive salt consumption surpassing WHO recommendations by fourfold or more (35). Consequently, there is a pronounced necessity for health education endeavors aimed at fostering healthier dietary choices.

Secondary prevention measures involve the implementation of routine screenings for CVD and DM2. While screening programs for conditions like arterial hypertension, ischemic heart disease, and DM2 are in operation across many Central Asian countries, they predominantly target individuals aged 40 years and older (15, 23, 32). Given our findings indicating an anticipated surge in CVD and DM2 burdens, deliberation on extending the coverage of these screening initiatives to younger population segments is imperative. Additionally, embracing innovative technologies such as telemedicine, mobile health applications, and wearable devices can enhance healthcare delivery, particularly in remote and geographically challenging mountainous regions (36) where a significant portion of the Central Asian population resides, thereby augmenting access to essential healthcare services. Regarding tertiary prevention strategies, they are largely established, as all Central Asian countries have instituted curative services for CVD, DM2, and stroke (35). Finally, but notably, an important potential impact of this study is that by extending preventive strategies for CVD and DM2 to women of reproductive age, improved patient satisfaction with the quality of healthcare services is likely to be achieved, a factor currently undermined in Central Asian countries (37).

In addition, it is paramount to integrate reproductive risk factors into cardiovascular risk assessments and national clinical guidelines to effectively mitigate potential risks and enhance health outcomes for women in their reproductive age. By incorporating certain reproductive risk into locally available cardiovascular risk assessment tools and national standards of care, healthcare providers can obtain a more comprehensive understanding of a woman’s cardiovascular health status and tailor interventions accordingly. For example, such pregnancy-related complications as gestational diabetes, preeclampsia, and gestational hypertension are established risk factors for future cardiovascular disease in women (38). Epidemiological studies indicate that they are prevalent in population of Central Asia (39–41). Including a thorough assessment of these complications in cardiovascular risk assessments allows healthcare professionals to identify individuals at heightened risk and implement early preventive measures (38).

Although the incidence of CVD is decreasing among older adults in many countries, there is evidence suggesting an increase in middle-aged individuals, with women aged 35–54 years experiencing the greatest rise in incidence cases (42). This trend can be attributed to various factors. On one hand, there is mounting evidence indicating that women of reproductive age are increasingly engaging in unhealthy behaviors, such as smoking and alcohol consumption. A longitudinal study conducted in Denmark suggests that these behaviors affect women more significantly than men (43, 44). On the other hand, women face specific fertility-related complications that do not affect men. For instance, research has shown that pregnancy-related complications like gestational hypertension and diabetes predispose women to develop arterial hypertension and DM2 post-pregnancy (38). Epidemiological data suggests that up to one third of parous women in developed countries experience at least one pregnancy-related complication, including gestational hypertension and diabetes (45). Furthermore, the risks associated with CVD and DM2 extend beyond pregnancy. Studies have demonstrated that women affected by conditions such as endometriosis and polycystic ovary syndrome are at a higher risk of developing ischemic heart disease and stroke (46, 47). Additionally, a longitudinal study conducted in Denmark indicates that the use of hormonal contraception is associated with an increased risk of stroke and myocardial infarction (48). Further research is warranted to fully elucidate these associations, in-form preventive strategies, and understand the extent to which they are relevant for countries in Central Asia.

Finally, the study’s relevance is amplified in the context of the COVID-19 pandemic, as it has highlighted the increased risk faced by patients with pre-existing conditions (49, 50). During the pandemic, individuals with chronic diseases were at a significantly higher risk of severe illness and mortality due to COVID-19 (51–53). The intersection of chronic diseases and infectious diseases like COVID-19 has exposed gaps in healthcare systems and emphasized the need for robust health policies that prioritize the management and prevention of chronic diseases. This pandemic has acted as a catalyst for health policy reform, urging governments in Central Asia to integrate chronic disease management into emergency preparedness plans. By doing so, healthcare systems can better protect vulnerable populations during health crises and ensure continuity of care for those with chronic conditions.

One primary limitation of our study pertained to the absence of direct access to data provided by national organizations for analysis. Furthermore, it would be beneficial to consider additional influencing factors beyond affiliation with the insurance sector, which could enhance comprehension of the subject matter.

## 5 Conclusion

In conclusion, the overarching trend across Central Asia reveals a significant increase in the prevalence of CVD, DM2, and stroke among women of reproductive age. Forecasts indicate a rising burden of these conditions, with growing YLL and YLD. Addressing these forecasts necessitates a multi-pronged approach, including strengthening healthcare systems, promoting research to understand country-specific risk factors, and fostering regional collaborations for knowledge exchange and resource pooling. Identifying reproductive risk factors during the early stages of women’s lives holds promise for initiating strategies to mitigate potential risks. Policy considerations should encompass the integration of reproductive risk factors into cardiovascular risk assessments and clinical guidelines. As these nations prepare for the upcoming decade, it is crucial to prioritize these health metrics, ensuring the well-being of women of reproductive age, a demographic pivotal for the region’s future.

## Data Availability

Publicly available datasets were analyzed in this study. This data can be found at: Institute for Health Metrics and Evaluation at: https://vizhub.healthdata.org/gbd-results/.
